# MOLER: Incorporate Molecule-Level Reward to Enhance Deep Generative Model for Molecule Optimization

**DOI:** 10.1109/tkde.2021.3052150

**Published:** 2021-01-21

**Authors:** Tianfan Fu, Cao Xiao, Lucas M. Glass, Jimeng Sun

**Affiliations:** Department of Computer Science and Engineering, Georgia Institute of Technology, Atlanta, GA 30332 USA.; Analytics Center of Excellence, IQVIA, Cambridge, MA 02139 USA.; Analytics Center of Excellence, IQVIA, Plymouth Meeting, PA 19462 USA, and also with Temple University, Philadelphia, PA 19122 USA.; Computer Science Department, University of Illinois, Urbana-Champaign, Champaign, IL 61820 USA.

**Keywords:** Automatic molecule optimization, generative models, molecule generation, drug discovery

## Abstract

The goal of molecular optimization is to generate molecules similar to a target molecule but with better chemical properties. Deep generative models have shown great success in molecule optimization. However, due to the iterative local generation process of deep generative models, the resulting molecules can significantly deviate from the input in molecular similarity and size, leading to poor chemical properties. The key issue here is that the existing deep generative models restrict their attention on substructure-level generation without considering the entire molecule as a whole. To address this challenge, we propose Molecule-Level Reward functions (MOLER) to encourage (1) the input and the generated molecule to be similar, and to ensure (2) the generated molecule has a similar size to the input. The proposed method can be combined with various deep generative models. Policy gradient technique is introduced to optimize reward-based objectives with small computational overhead. Empirical studies show that MOLER achieves up to 20.2% relative improvement in success rate over the best baseline method on several properties, including QED, DRD2 and LogP.

## 1 Introduction

Designing molecules with desirable properties is a fundamental task in drug discovery. Traditional methods such as high throughput screening (HTS) tests large compound libraries to identify molecules with desirable properties, which are inefficient and costly [1], [2]. Unlike HTS, *molecule optimization* takes an input molecule *X* and aims to find molecule *Y* with improved drug properties such as drug-likeness and biological activity. However, standard molecule optimization is still costly and time-consuming due to the long cycles of labor-intensive synthesis and analysis [3].

The challenges mentioned above motivate a series of works that apply machine learning techniques on molecule optimization, with the ultimate goal to generate new molecules that *maximize desirable drug properties while maintaining similarity* to the original molecule. Existing works mainly can be categorized as generative models [4], [5], [6] and reinforcement learning (RL) methods [7], [8]. *Generative models for molecule optimization* map an input molecule to a latent space and perform optimization in the latent space to search for new molecules. For example, Gómez-Bombarelli *et al.* [6], Blaschke *et al.* [9] utilized SMILES strings as molecule representations to generate molecules; however, they are known to create many invalid molecules. To improve the validity of the generated molecules, various approaches were proposed: Kusner *et al.* [4] and Dai *et al.* [5] designed grammar constraints, while MolGAN [10], CGVAE (Constrained Graph VAE) [11], JTVAE (Junction Tree VAE)-based approaches [12], [13], [14] focus on graph representations of molecules. *Reinforcement learning for molecule optimization* are often developed on top of molecule generators for optimizing desirable properties. For example, Olivecrona *et al.* [15], Putin *et al.* [16], and Popova *et al.* [17] applied RL techniques on top of a string generator to produce SMILES strings. You *et al.* [7], Zhou *et al.* [8] proposed to generate molecular graphs, which achieved perfect validity.

**Fig. 1.**
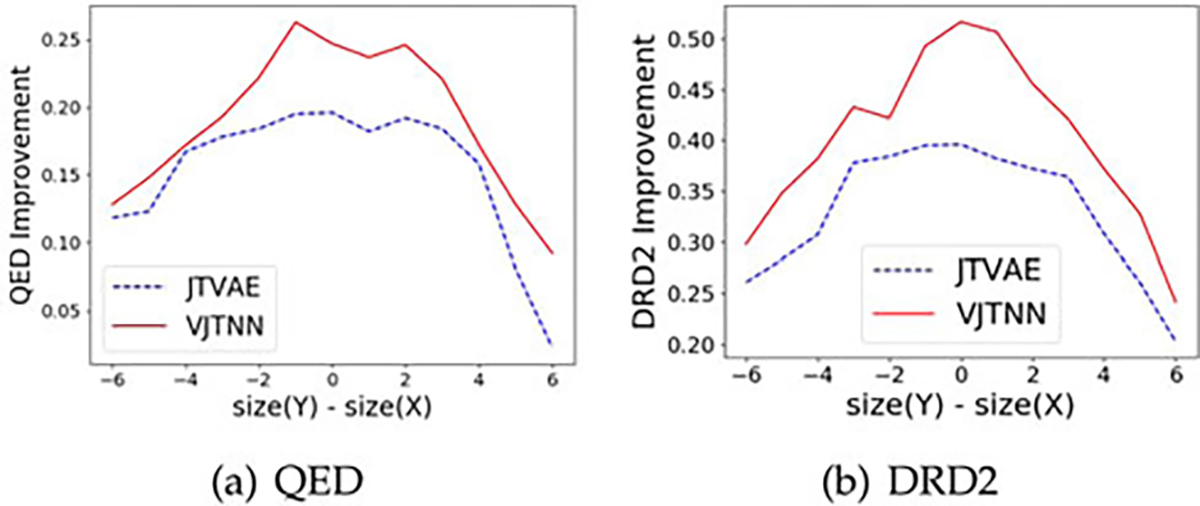
Property Improvement as a function of size difference between source molecule *X* and target molecule *Y*.

Despite the initial success, existing methods often rely on iterative local expansion to acquire the target molecule, potentially creating molecules of arbitrary sizes. Without a global fitness metric, the generated molecules can significantly deviate from the target molecule in molecular similarity and size. Also, these methods focus on patterns that map on substructures. However, during testing, the evaluation metrics for molecule quality are often based on the entire molecule. These discrepancies cause two challenges. We also empirically demonstrate them using Junction Tree Variational Auto-Encoder (JTVAE) [12] and Variational Junction Tree encoder-decoder (VJTNN) [13] models, as shown in Table 1 and Fig. 1.

- *Difficulty in Maintaining Similarity While Optimizing Drug Properties*. Similarity scores between input and generated molecules (defined in Equation (11)) are relatively low compared with property improvement. For example, when property scores are higher than 0.7 in the range [0,1], the similarity score will only be low (around 0.3 in the range [0,1]), as shown in Table 1.
- *Difficulty in Maintaining Molecule Sizes Which Affect Property Improvement*. When generated molecules are of very different sizes compared to the target molecules, they will have poor performance in both similarity and property. Fig. 1 show the property improvement as a function of the size difference between the generated and input molecules. We can see that a large size difference in either positive or negative direction usually corresponds to smaller property improvement.

To address these challenges, we introduce Molecule-level Rewards (MOLER) to enhance the molecule level properties. MOLER is a general and flexible approach that can be incorporated into various deep generative models for improved performance. MOLER is enabled by the following technical contributions.

- *Similarity Reward* (Section 3.2): We formulate the similarity between input and generated molecules as a reward function to alleviate the *similarity gap* between them.
- *Size deviation penalty* (Section 3.3): To explicitly control the size of molecule, we design a size deviation penalty that penalizes large size deviations to reduce the *size difference* between target and generated molecules.

We evaluated MOLER by incorporating it into JTVAE [12], VJTNN [13] and CORE [18]. We compared the MOLER-enhanced models with several strong baselines on three real-world molecule optimization tasks. Results show MOLER-enhanced models can achieve up to 16%, 14%, and 20% relatively improvement over the best baselines on similarity score, property improvement, and success rate, respectively.

## 2 Related Works

We review related works in molecule generation. From the methodology perspective, there are two research lines in automatic molecule generation, namely the maximum likelihood estimation (MLE) via deep generative models (DGM), and the reinforcement learning (RL)-based approaches.

*Maximum Likelihood Estimation (MLE) methods* include Sequence-to-Sequence (Seq2Seq) models and graph-to-graph ones. The Seq2Seq models focus on maximizing the likelihood of the target sequence and is proven effective in various natural language processing tasks [19]. Since molecule can be also represented as SMILES sequences, it is straightforward to apply the Seq2Seq model. Sequence-based approaches are very brittle since small changes to the sequence may completely violate chemical validity. Thus special constraints were developed to ensure the validity of generated SMILES string [4], [5], [6]. However, due to the limited representation power of SMILES and complex syntactic constraints on SMILES string, these sequence-based approaches still suffer from generating invalid SMILES string. To address this issue, graph-based methods were explored, where graph representation of molecules can capture the structural information of molecules. Specifically, [12] represented molecule graph where nodes are substructures such as rings and atoms, and then applied a two-phase generation procedure to create a scaffolding tree. The generation is to assemble the tree into a new molecular graph. More recently, the graph-to-graph translation model [13] was proposed as an enhancement of [12]. It extends the junction variational autoencoder via attention mechanism and generative adversarial networks. One potential problem for MLE methods often requires paired training data (i.e., input and target molecule pairs), which are not naturally available for molecule optimization.

*Reinforcement Learning (RL) methods*were also adopted in sequence-based molecule generation [15], [20] and graph-based generation [7], [8]. The learning objective is based on molecule level reward, e.g., directly introducing chemical property of the generated molecule as reward. However, RL-based methods usually ignore substructure-level supervision and suffer from poor exploration efficiency [21] and are usually hard to train [13], [22].

Between the two, the MLE/DGM methods focus on substructure-level translation and ignore the molecule-level reward [12], [13], [18], [23]. Also, MLE methods train on paired molecules, which are not naturally available for molecule optimization and thus limit the performance. In contrast, for RL methods, the learning objective is based on molecule-level reward, e.g., directly introduce the chemical property of the generated molecule or molecular similarity as reward. RL-based methods usually ignore substructure-level supervision and suffer from poor exploration efficiency [21] and are generally hard to train [13], [22]. To alleviate these issues, our method bridges the two approaches by incorporating two molecule-level rewards into MLE/DGM models.

## 3 MOLER Method

In this section, we will first present deep generative models as background for molecule optimization in Section 3.1, particularly the scaffolding tree^1^ based graph generation approaches [12], [13], [14], [18].

**Fig. 2.**
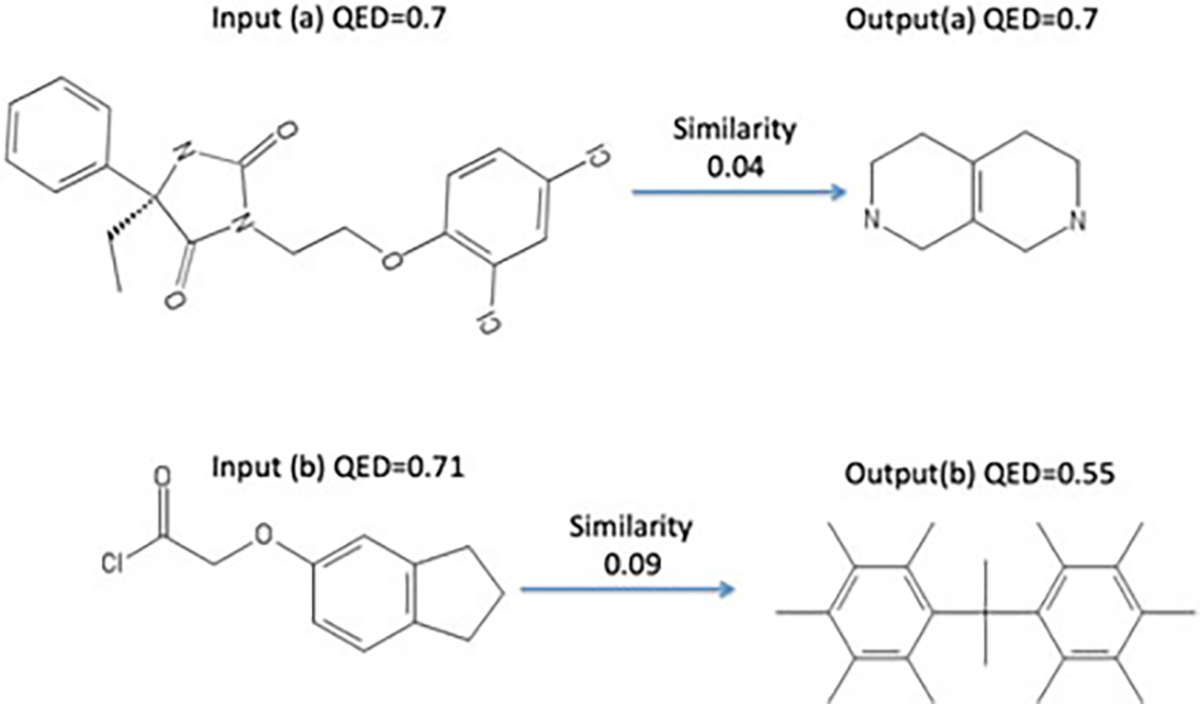
MOLER Framework. (1) First we describe the generative model. A molecular graph is transformed into a scaffolding tree. Both of them are encoded. Then, tree decoder and graph decoder are applied one after another. During the tree decoder, topological prediction generates the structure of scaffolding tree, while substructure prediction is to predict the substructure for the label of each node in the scaffolding tree. After that, during the graph decoder, scaffolding tree is assembled into molecules. SGD is applied to optimize generative models. (2) Then in MOLER, we use the generative model to generate molecules and evaluate their reward, where the two reward functions (similar reward and size deviation penalty) will assign high reward to the molecule that is (a) similar to input molecule *X*; (b) close to *X* in molecular size. Policy gradient is used to optimize the reward.

After introducing the background on scaffolding tree-based generative model and its limitations, we present the two molecule reward functions individually in Sections 3.2 and 3.3 followed by the optimization procedure (Section 3.4). We list some important mathematical notations and their short explanations in Table 2 for clarity, and also show a flow chart in Fig. 2 to illustrate deep generative models and MOLER in an intuitive way.

### 3.1 Deep Generative Models for Molecule Generation

In this subsection, we summarize the scaffolding tree-based generative model for molecule generation, which achieves the state of the art performance [12], [13], [14], [18]. For ease of exposition, we first define molecular graph, substructure and scaffolding tree. Note that this subsection provides the background to understand these types of generative models, which were originally proposed in [12] then subsequently enhanced by [13], [14], [18]. The main contribution of this paper will be discussed in next subsection.

#### Definition 1 (Molecular Graph *G*).

Molecular graph G represents the graph structure for a molecule. In molecule graph G, each node corresponds to an atom of a molecule, and each edge corresponds to a bond connecting atoms.

#### Definition 2 (Substructure).

The set of substructures includes all possible rings, bonds, and atoms. A substructure is a basic unit in the scaffolding tree. Each substructure corresponds to a node in the scaffolding tree.

#### Definition 3 (Scaffolding Tree ${𝒯}_{G}$).

Given a molecule and its corresponding molecular graph G, the scaffolding tree 𝒯 is generated by partitioning the graph G into substructures and connecting substructures into a tree. The main purpose of using scaffolding trees is to simplify the generation procedure. The scaffolding tree ${𝒯}_{G}$ is the skeleton of the molecular graph.

These models for generating valid molecules comprise of two parts: (1) the encoder-decoder for scaffolding tree ${𝒯}_{G}$, and 2) the encoder-decoder for molecular graph *G*.

For scaffolding tree-based graph generation methods, the training set consists of paired data (*X*, *Y*). The input is molecule *X* while the target is another molecule *Y* similar to *X* but with better chemical property, e.g., biological activity (measured by DRD) [24], drug-likeness (measured by QED) [25]. The neural architecture can be divided into two parts: (i) encoder and (ii) decoder. Both encoder and decoder have molecular graph phase and scaffolding tree phase.

#### 3.1.1 Encoder

The goal of the encoder is to yield an embedding vector for each node in the scaffolding tree ${𝒯}_{G}$ or the input molecular graph *G*. Specifically, both input molecular graphs and scaffolding trees are encoded via graph Message Passing Networks (MPN) [12], [26]. It encodes both nodes and edges in a graph. First, on the node level, each node *v* has a feature vector denoted **e**_*v*_. For example, node *v* in a molecular graph *G* is an atom, **e**_*v*_ includes the atom type, valence, and other atomic properties. In the corresponding scaffolding tree ${𝒯}_{G}$, node *v* refers to a substructure, then **e**_*v*_ is a one-hot vector indicating the substructure’s index. On the edge level, **e**_*uv*_ is the feature vector for edge (*u; v*) ∈ *E*. *N*(*u*) represents the set of all the neighbor nodes of the node *u*. **m**_*uv*_ and **m**_*vu*_ are the hidden variables representing the message from node *u* to node *v* and vice versa. They are iteratively updated as

(1) $$m_{uv}^{\left(t\right)}=f_{1}\left(e_{u},e_{uv},\sum_{w\in N(u)\backslash v}m_{wu}^{\left(t-1\right)}\right),\;t=1,\ldots ,T,$$

where *f*_1_(·) is a fully-connected neural network, $m_{uv}^{\left(t\right)}$ is the message vector from node *u* to node *v* at the *t*th iteration, whose initialization is all-0 vector, i.e., $m_{uv}^{\left(0\right)}=0$, following the rule of thumb [26]. After *T* steps of iteration,^2^ another fully-connected neural network *f*_2_(·) is used to aggregate these messages. Each vertex has a latent vector as

(2) $$z_{u}=f_{2}\left(e_{u},\sum_{v\in N(u)}m_{vu}^{\left(T\right)}\right).$$

In a nutshell, the encoder module yields embedding vectors for nodes in graph *G* and scaffolding tree ${𝒯}_{G}$, denoted $Z^{G}=\left\{z_{1}^{G},z_{2}^{G},\ldots \right\}$ and $Z^{{𝒯}_{G}}=\left\{z_{1}^{{𝒯}_{G}},z_{2}^{{𝒯}_{G}},\ldots \right\}$, respectively.

#### 3.1.2 Decoder

Like encoder, molecule generation can be also divided into two parts: A. (scaffolding) tree decoder; B. (molecular) graph decoder. The goal of the scaffolding tree decoder is to generate a new scaffolding tree. Then graph decoder converts the scaffolding tree into the correct molecular graph.

##### A. Tree Decoder.

To generate a new scaffolding tree, the idea is to generate one substructure at a time from an empty tree. In each step, first it needs to decide/predict whether to expand the current node or backtrack to its parent (*topological prediction*). If the prediction is to expand, (*substructure prediction*) is leveraged to predict which substructure to add to the new node, as shown in Fig. 2. When it backtracks to the starting node and decide not to expand any more, the generation process terminates automatically.

##### Topological Prediction.

The core idea is first leveraging tree-based Recurrent Neural Network (tree-RNN) [12] to enhance the embedding for node *i*_*t*_, then predict whether to expand or backtrack. Given scaffolding tree ${𝒯}_{G}=(𝒱,ℰ)$, the tree decoder uses the tree-based RNN with attention mechanism to further improve embedding information learned from the original message-passing embeddings $Z^{𝒯}$. The tree-RNN converts graph into a sequence of nodes and edges via depth-first search. Specifically, let $\tilde{ℰ}=\left\{\left(i_{1},j_{1}\right),\left(i_{2},j_{2}\right),\ldots ,\left(i_{m},j_{m}\right)\right\}$ be the edges traversed in depth first search, each edge is visited twice in both directions, so we have $m=|\tilde{ℰ}|=2|ℰ|$. Suppose ${\tilde{ℰ}}_{t}$ is the first *t* edges in $\tilde{ℰ}$, message vector $h_{i_{t},j_{t}}$ is updated as

(3) $$h_{i_{t},j_{t}}=\mathrm{GRU}\left(e_{i_{t}},{\left\{h_{k,i_{t}}\right\}}_{\left(k,i_{t}\right)\in {\tilde{ℰ}}_{t},k\neq j_{t}}\right).$$

The expanding probability at node *i*_*t*_ is computed by combining the embeddings $Z^{𝒯}$, **Z**^*G*^ and the current state $e_{i_{t}}$, $\sum_{\left(k,i_{t}\right)\in {\tilde{ℰ}}_{t}}h_{k,i_{t}}$ using a neural network *f*_3_(·)

(4) $$p_{t}^{\text{topo}}=f_{3}\left(e_{i_{t}},\sum_{\left(k,i_{t}\right)\in {\tilde{ℰ}}_{t}}h_{k,i_{t}},Z^{𝒯},Z^{G}\right),\;\text{where}\;t=1,\ldots ,m.$$

Specifically, we first compute context vector $c_{t}^{\text{topo}}$ using attention mechanism (similar to the process in Equations (5) and (6)), then concatenate $c_{t}^{\text{topo}}$ and $e_{i_{t}}$, and feed into a fully-connnected neural network with sigmoid function to produce the expanding probability.

##### Substructure Prediction.

After the decision of node expansion, we focus on finding what substructures to add by either selecting from the global set of substructures [12], [13] or copying from original input [18]. Every time after expanding a new node, the model predicts the substructure from the vocabulary.

We will summarize the key steps next. First we use attention mechanism to compute context vector based on current message vector $h_{i_{t},j_{t}}$ and node embedding $Z^{𝒯}$, **Z**^*G*^

(5) $$c_{t}^{\text{sub}}=\mathrm{Attention}\left(h_{i_{t},j_{t}},Z^{𝒯},Z^{G}\right),$$

Specifically, the attention weight is computed as

(6) $$\alpha_{j}^{𝒯}=f_{4}\left(h_{k,i_{t}},z_{j}^{𝒯}\right),\;\alpha_{i}^{𝒯}\in \mathbb{R},\;\left[\alpha_{1}^{𝒯},\alpha_{2}^{𝒯},\ldots \right]=\mathrm{Softmax}\left(\left[\alpha_{1}^{𝒯},\alpha_{2}^{𝒯},\ldots \right]\right),$$

where *f*_4_(·) is the dot-product function [28]. {*α*^*G*^) are generated in the same way. Then we concatenate tree-level and graph-level context vector to generate the context vector

(7) $$c_{t}^{\text{sub}}=\left[\sum_{i}\alpha_{i}^{𝒯}z_{i}^{𝒯},\sum_{j}\alpha_{j}^{G}z_{j}^{G}\right].$$

Then based on attention vector $c_{t}^{\text{sub}}$ and message vector $h_{i_{t},j_{t}}$, *f*_5_(·), a fully-connected neural network with softmax activation, is added to predict the substructure

(8) $$q_{t}^{\text{sub}}=f_{5}\left(h_{i_{t},j_{t}},c_{t}^{\text{sub}}\right),$$

where $q_{t}^{\text{sub}}$ is a probability distribution over all substructures [12], [13]. In [18], $q_{t}^{\text{sub}}$ is enhanced by a COPY strategy that copies substructures from the input molecule using attention weight in Equation (6).

##### B. Graph Decoder.

Based on the scaffolding tree generated by the tree decoder, graph decoder is used to convert nodes in the scaffolding tree together into the correct molecular graph [12]. During the learning procedure, we enumerate all the possible molecular structures {*G*_*i*_}. Then we formulate it as a multi-classification problem, where the learning objective is the log-likelihood of the right structure *G*_*o*_

(9) $${ℒ}_{g}=s^{a}\left(G_{o}\right)-\log\sum_{G_{i}}\exp\left(s^{a}\left(G_{i}\right)\right),$$

where $s^{a}\left(G_{i}\right)=h_{G_{i}}^{⊤}d_{G_{o}}$ is a scoring function that measures the likelihood of the current structure *G*_*i*_; $d_{G_{o}}$ is the embedding of the original graph *G*_*o*_.

##### Limitation and Extensions.

The problem of these generative models is that they only focus on the local (substructure level) mapping while ignore the global (molecule level) metric, which may lead a significant deviation from input molecule in similarity. Next, we will present RL-based reward functions to enhance the overall loss function towards more desirable molecules.

### 3.2 Similarity Reward

Both similarity with input molecule *X* and property of *Y* (denoted sim(*X*, *Y*)) are essential metrics to evaluate the quality of generated *Y*. The similarity between two molecules ranges from 0 to 1. As mentioned, the similarity value is relatively low (around 0.3 for both QED and DRD2 dataset) compared with property value (approximately 0.7–0.8) in numerical value. We consider adding “similarity reward” to explicitly enhance the similarity constraint, i.e., maximize

(10) $${ℒ}_{\mathrm{sim}}(\theta )=E_{Y\sim \pi_{\theta }(\cdot |X)}[\mathrm{sim}(X,Y)],$$

where *θ* represents all the parameters for generative models; ${ℒ}_{\mathrm{sim}}(\theta )$ is objective function for similarity reward; hyperparameter $w_{\text{sim}}\in {\mathbb{R}}_{+}$ is weight of the reward. sim(*X*, *Y*) represents Tanimoto similarity between molecule *X* and *Y* over Morgan fingerprints [29]. Morgan fingerprints is a binary vector where each bit records the presence (“1”) or absence (“0”) of a binary fragment descriptor in the molecule. For example, a fragment descriptor can be “if Benzene ring exists in the molecule”. It is assumed that two fingerprints with many bits in common represent similar molecules. Let ${𝒮}_{X}$ and ${𝒮}_{Y}$ denote the set of fragment descriptors that exist in molecule X and Y, respectively (i.e., presence of fragment descriptor). Tanimoto similarity is defined as

(11) $$\mathrm{sim}(X,Y)=\frac{\left|{𝒮}_{X}\;\cap \;{𝒮}_{Y}\right|}{\left|{𝒮}_{X}\;\cup \;{𝒮}_{Y}\right|}\in [0,1],$$

where ∩, ∪ represent the intersection and union of two binary vectors respectively; |·| denotes the cardinality of a set. The value ranges from 0 to 1. Higher value means more similarity. We use Rdkit package [30] for both generations of Morgan fingerprints and computation of Tanimoto similarity. When optimizing Equation (10), the computation of its gradient estimator requires the numerical value of the probability density function *π*_*θ*_(*Y*|*X*) explicitly. Now we discuss how to evaluate *π*_*θ*_(*Y*|*X*) explicitly.

We know from Section 3.1, the molecule generation is mainly divided into three prediction tasks: (i) topological prediction, which is a binary classification task; (ii) substructure prediction; (iii)Assembling prediction (in graph decoder). Since there are two phases (scaffolding tree and molecular graph) in graph generation, *π*_*θ*_(*Y*|*X*) can be written as the following joint distribution that incorporate the generation of both the scaffolding tree and the molecular graph, which includes three prediction tasks,

(12) $$\begin{array}{l} \pi_{\theta }(Y|X)\\ =\underset{\text{topological}\;\text{prediction}}{\underset{︸}{\prod_{t=1}^{2|ℰ|}p_{t}^{\text{topo}}}}\cdot \underset{\text{substructure}\;\text{prediction}}{\underset{︸}{\prod_{t\in 𝒮}q_{t}^{\text{sub}}}}\;\;\;\;\;\;\cdot \underset{\text{assembling}\;\text{prediction}}{\underset{︸}{\frac{\exp\left(s^{a}\left(G_{Y}\right)\right)}{\sum_{G_{i}}\exp\left(s^{a}\left(G_{i}\right)\right)}}}. \end{array}$$

First, $p_{t}^{\text{topo}}$ is the probability for the *t*th topological prediction, defined in Equation (4). ℰ is edge set of the generated scaffolding tree. Since the generation procedure would terminate until backtracking to root node, each edge is visited twice and there are totally 2|ℰ| topological predictions. Second, regarding substructure prediction, $q_{t}^{\text{sub}}$ is a distribution over all substructures defined Equation (8), 𝒮⊆{1,2,…,2|ℰ|} represents the set of the indexes of the edges who expands to a new node in a scaffolding tree, because only when topological prediction $p_{t}^{\text{topo}}$ predict to expand to a new node, we need to make substructure prediction. Third, regarding assembling prediction, *G*_*Y*_ is the assembling structure for *Y*, selected from all the possible molecular structure {*G*_*i*_} based on the scaffolding tree, $s^{a}\left(G_{i}\right)=h_{G_{i}}\cdot d_{G_{o}}$ is a scoring function defined in Equation (9).

### 3.3 Size Deviation Penalty

Size deviation between input and target molecules is another reason that the target molecule is dissimilar to the input. Therefore, we design a penalty score to constrain this deviation.

As mentioned in Section 3.1, in graph generation there are three key prediction tasks: (i) topological prediction; (ii) substructure prediction; (iii) assembling prediction. Since scaffolding tree-based approaches use substructure as the basic component in molecule generation, we use the number of substructure as the surrogate for molecule size, which is much more efficient to compute since it is only related to topological prediction.

To design a reasonable size deviation penalty, we empirically investigate the correlation between the size of *X* and *Y* in training data pairs, and find they are positively correlated. We want to minimize the following size deviation penalty,

(13) $${ℒ}_{\text{size}}(\theta )=E_{\pi_{\theta }^{T}\left(T_{Y}|X\right)}\left[g\left(\mathrm{size}\left(T_{X}\right),\mathrm{size}\left(T_{Y}\right)\right)\right],$$

where ${ℒ}_{\text{size}}(\theta )$ is objective function of size deviation penalty, hyperparameter $w_{\text{size}}\in {\mathbb{R}}_{+}$ is weight for size deviation penalty, size(*X*) denotes the number of substructure in scaffolding tree of *X*. *g*(·, ·) is the reward function of molecule size defined as

(14) $$g(x,y)=\{\begin{array}{ll} |x-y|-\epsilon , & |x-y|>\epsilon ,\\ 0, & |x-y|\leq \epsilon , \end{array}$$

where $\epsilon \in {\mathbb{N}}_{+}$ is a positive integer. We set *ϵ* = 3, which is empirically validated. Note that *g*(·, ·) is commonly known as *ϵ*-insensitive loss function in the context of SVM regression [31]. The intuition behind the design of *g*(*x*, *y*) is to give a high penalty when the size of the input and generated molecule differs significantly. When minimizing ${ℒ}_{\text{size}}(\theta )$, we need to evaluate $\pi_{\theta }^{T}$ explicitly, which is a joint distribution for all topological prediction,

(15) $$\pi_{\theta }^{T}(Y|X)=\prod_{t=1}^{2|ℰ|}p_{t}^{\text{topo}}.$$

### 3.4 Learning and Optimization

Now we discuss the optimization procedure. Without loss of generalization, we consider maximizing a general objective as follows,

(16) $$ℒ(\theta )=E_{Y\sim \pi_{\theta }(\cdot |X)}[R(X,Y)],$$

where ℒ(θ) can be either ${ℒ}_{\text{sim}}$ or ${ℒ}_{\text{size}}$ in this paper, reward function R(X,Y)∈ℝ can be either sim(*X*, *Y*) or *g*(size(*X*), size(*Y*)). *R*(*X*, *Y*) usually doesn’t involve *θ*, so it’s seen as a constant if we optimize w.r.t. *θ*. $\pi_{\theta }(Y|X)\in {\mathbb{R}}_{+}$ corresponds the probability density function for generative model.$\nabla_{\theta }ℒ(\theta )$, the gradient of objective function with regard to *θ*, can be expanded as

(17) $$\nabla_{\theta }ℒ(\theta )=\nabla_{\theta }\int \pi_{\theta }(Y|X)R(X,Y)dY\;\;=\int \nabla_{\theta }\pi_{\theta }(Y|X)R(X,Y)dY\;\;=\int \pi_{\theta }(Y|X)\nabla_{\theta }\log\pi_{\theta }(Y|X)R(X,Y)dY\;\;=E_{Y\sim \pi_{\theta }(\cdot |X)}\left[\nabla_{\theta }\log\pi_{\theta }(Y|X)R(X,Y)\right],$$

where in the third equality, “log-derivative trick” is applied. It is a well-known technique in policy gradient-based reinforcement learning [32], [33] and stochastic variational inference [34]. Then, the unbiased estimator for gradient of ℒ(θ) with regard to parameters *θ* can be obtained using Monte Carlo samples,

(18) $$\nabla_{\theta }ℒ(\theta )\approx \frac{1}{m}\sum_{i=1}^{m}\nabla_{\theta }\log\pi_{\theta }\left({\tilde{Y}}_{i}|X_{i}\right)R\left(X_{i},{\tilde{Y}}_{i}\right),$$

where ${\tilde{Y}}_{i}\sim \pi_{\theta }\left(\cdot |X_{i}\right)$. That is, ${\tilde{Y}}_{i}$ is a molecule generated by generative model given the input molecule *X*_*i*_. Then gradient ascent is used to maximize the objective function with regard to *θ*. Worth to mention that the proposed method does not require extra parameters for the model compared with the deep generative models.

In summary, the deep generative model and two reward-based objectives are optimized alternatively and individually. Stopping criteria is checked every epoch on the validation set. When the success rate (a metric, which would be discussed in Section 4.5) of the current epoch is lower than the previous epoch, we terminate the learning procedure. We show the complete algorithm in Algorithm 1, which has more details.

**Algorithm 1.** MOLER

1:	# training
2:	**while** Convergence criteria is not met **do**
3:	Sample a minibatch $ℳ=\left\{\left(X_{1},Y_{1}\right),\ldots ,\left(X_{m},Y_{m}\right)\right\}$, m=|ℳ|
4:	Optimize ${ℒ}_{\text{gen}}(\theta )$ w.r.t. *θ* using ${\left\{\left(X_{i},Y_{i}\right)\right\}}_{i=1}^{m}$ using SGD, where ${ℒ}_{\text{gen}}(\theta )$ is loss of generative models.
5:	Generate molecule via ${\tilde{Y}}_{i}\sim \pi_{\theta }\left(\cdot |X_{i}\right)$ for *i* = 1, …, *m*.
6:	Maximize ${ℒ}_{\text{sim}}(\theta )+{ℒ}_{\text{size}}(\theta )$ w.r.t. *θ* using ${\left\{\left(X_{i},{\tilde{Y}}_{i}\right)\right\}}_{i=1}^{m}$ based on gradient estimator in Equation (18).
7:	**end while**
8:	# test, e.g., QED task
9:	**for** *X_i_* ∈ Test Set **do**
10:	generate *Y_i_* ~ *π_θ_*(·|*X_i_*).
11:	Evaluate and record sim(*X_i_*, *Y_i_*) and QED(*Y*) – QED(*X*)
12:	**end for**
13:	Evaluate average similarity, property improvement and success rate on the whole test set.

### 3.5 Choosing Weight for MOLER

During learning procedure, two reward values (related to similarity and size) are integrated in the learning objective in order to minimize

(19) $$ℒ={ℒ}_{\text{gen}}-w_{\text{sim}}{ℒ}_{\text{sim}}+w_{\text{size}}{ℒ}_{\text{size}},$$

where *w*_sim_, $w_{\text{size}}\in {\mathbb{R}}_{+}$ are hyperparameters that control the strength of MOLER, ${ℒ}_{\text{gen}}$ is loss of generative model. It is time-consuming to use grid search/random search to find the near-optimal weight combination (*w*_*sim*_, *w*_*size*_). To address this issue, we resort to Gaussian process (GP) [35], [36]. Gaussian process is used to approximate the validation accuracy as a function of these weight combination. The validation accuracy can be success rate, which is described in Section 4.5) Then via searching for the optimum of the approximated function, we obtain the appropriate weight combinations. Concretely, we denote the function to approximate as *h*(*w*_1_, *w*_2_), where *w*_1_ = *w*_sim_, *w*_2_ = *w*_size_. The search range for *w*_*i*_, *i* = 1, 2 is bounded by LB_*i*_ ≤ *w*_*i*_ ≤ UB_*i*_. We draw *m* weight combinations, denoted $w^{\left(1\right)}=\left(w_{1}^{1},w_{2}^{1}\right)$;$w^{\left(2\right)}=\left(w_{1}^{2},w_{2}^{2}\right);\ldots ;w^{\left(m\right)}=\left(w_{1}^{m},w_{2}^{m}\right)$. For the *i*th weight combination **w**^(*i*)^, we evaluate the task metric (e.g., success rate) on validation set *r*^*i*^, which can be seen as a noisy evaluation of the true function $h\left(w_{1}^{i},w_{2}^{i}\right)$ we want to approximate. The noise comes from sampling bias of validation set. The true objective function *h*(*w*_1_, *w*_2_) is unknown, a zero-mean Gaussian prior is specified,

(20) h(⋅)~GP(⋅,k(⋅,⋅)),

where *k*(·, ·) is the covariance function. Given *m* points ${\left\{w^{\left(i\right)}\right\}}_{i=1}^{m}$ (weight combination) and their evaluation ${\left\{r^{i}\right\}}_{i=1}^{m}$. We assume *r* is a noisy evaluation of the true unknown function that we are interested, i.e., *r*(**w**) = *h*(**w**) + *ϵ*, **w** = (*w*_1_, *w*_2_). According to Bayes rule, the posterior distribution of the true objective function is

(21) $$h|{\left\{w^{\left(i\right)},r^{i}\right\}}_{i=1}^{m}\sim 𝒩\left(\mu (w),\sigma^{2}(w)\right),\;\mu (w)=k^{⊤}{\left(K+\sigma^{2}I\right)}^{-1}r,\;\sigma (w)=k(w,w)-k^{⊤}{\left(K+\sigma_{n}^{2}I\right)}^{-1}k,$$

where

(22) $$\begin{array}{l} K=\left[\begin{array}{ccc} k\left(w_{1},w_{1}\right) & \cdots & k\left(w_{1},w_{m}\right)\\ \vdots & \ddots & \vdots \\ k\left(w_{m},w_{1}\right) & \cdots & k\left(w_{m},w_{m}\right) \end{array}\right],\\ k={\left[k\left(w,w_{1}\right),\ldots ,k\left(w,w_{m}\right)\right]}^{⊤},\\ r={\left[r_{1},\ldots ,r_{m}\right]}^{⊤}. \end{array}$$

Regarding covariance function we set $k(w,v)=\exp\left(-\frac{1}{2}{\left(w-v\right)}^{⊤}\Sigma^{-1}(w-v)\right)$, where $\Sigma =\mathrm{diag}(\alpha {\left({\text{UB}}_{1}-{\text{LB}}_{1}\right)}^{2},\alpha {\left({\text{UB}}_{2}-{\text{LB}}_{2}\right)}^{2})$. *α* is empirically set to 0.2 [36].

To summarize, Gaussian process provides a surrogate function to approximate the true objective *h*(*w*_1_, *w*_2_), the validation accuracy as a function of weights in MOLER (*w*_sim_ and *w*_size_). The surrogate can be used to search, efficiently, for the optimum of the objective function, i.e., optimal weights in MOLER. Grid search is leveraged here to explore the surrogate and get the optimal weights.

## 4 Experiment

The experiment section answers the following questions.

*Q1:* Can MOLER improve deep generative models for molecular optimization?

*Q2:* What is the effect of similarity reward alone?

*Q3:* What is the effect of size deviation penalty alone?

### 4.1 Molecular Properties

We are usually interested in some desired chemical properties of molecule in drug discovery, which are natural metrics for evaluation. Following [7], [8], [13], [14], [23], [37], we also focus on the following molecular properties:

- *Quantitative Estimate of Drug-likeness (QED)*. QED is an indicator of drug-likeness [25], and the QED score of a compound ranges from 0 to 1.
- *Dopamine Receptor (DRD2)*. DRD2 score measures a molecule’s biological activity against a biological target named the dopamine type 2 receptor (DRD2) [24]. DRD2 score ranges from 0 to 1, and a high score means better property.
- *Penalized LogP*. Penalized LogP is a logP score that also accounts for ring size and synthetic accessibility [38]. LogP score ranges from −∞ to +∞. For most molecules, the score ranges from −20 to 20.

For any chemically valid molecule, QED, DRD2, and LogP scores can be evaluated via Rdkit package [30]. For all these three scores, a higher score is better. Thus, for the training data pairs (*X*, *Y)*, *X* is the molecule with lower scores, while *Y* is a paraphrase of *X* with a higher score.

### 4.2 Setup

First, we describe the experimental setup, including the chemical properties of molecules and the generation of training data. The molecular properties include Drug likeness (QED), Dopamine Receptor (DRD2), and Penalized LogP.

#### Dataset.

Data pairs of input and target molecules are extracted from the ZINC dataset. ZINC contains 250K drug molecules, which are extracted from the ZINC database.^3^

We use 3 public datasets publicly available in [13], including (1) QED dataset for improving QED; (2) DRD2 dataset for improving DRD2; (3) LogP dataset for improving Penalized LogP. In this paper, QED, DRD2, LogP are both the target chemical properties to improve and the name of datasets, e.g., QED dataset is used to learn a model for improving the QED score of a molecule. Some basic statistics of all the dataset are listed in Table 3.

### 4.3 Dataset Construction

The training datasets contain data pairs, which are extracted from ZINC dataset^4^ based on following two criteria:

- *Simlarity Constraint*. *X* and *Y* are similar enough, i.e.,
(23) $$\mathrm{sim}(X,Y)\geq \eta_{1}.$$
  Following [13], [23], [37], for all the three datasets, *η*_1_ = 0.4.
- *Property Constraint*. *Y* has a higher score than *X* on a certain property. For QED dataset, QED score of *X* are in the range of [0.7,0.8] while QED score of *Y* is in the range of [0.9,1]. For DRD2 dataset, a well-trained model in [40] is applied to assess the probability of biological activity, where probability of *X* is lower than 0.05 and the probability for *Y* is greater than 0.5. The property constraint for LogP dataset is described in [13], [39]. The settings are the same as [13].

### 4.4 Baseline Methods

Now we briefly introduce the baseline methods.

- *JTVAE* (Junction Tree Variational Auto-Encoder) [12].
- *VJTNN* (Variational Junction Tree Encoder-Decoder, also called graph-to-graph translation) [13].
- *CORE* (VJTNN with Copy and Refine Strategy) [18].
- *GCPN* (Graph Convolutional Policy Network) [7]. GCPN is state-of-the-art reinforcement learning-based method.
- *ReLeaSE*. Reinforcement Learning for Structural Evolution (ReLeaSE) [20] exhibits state-of-the-art performance on SMILES representation.

MOLER can be applied to enhance deep generative models such as JTVAE, VJTNN, and CORE.

### 4.5 Evaluation Metrics

Next, we provide some key metrics when evaluating the effectiveness of the generated molecules. The task is to generate *Y*, a paraphrase molecule of *X*, with better desired property. Here are the evaluation metrics used in our experiments:

- *Similarity*. We evaluate the molecular similarity between the input molecule and the generated molecule, measured by Tanimoto similarity over Morgan fingerprints [29], defined in Equation (11). Similarity between X and Y (denoted sim(*X*, *Y*)) ranges from 0 to 1. Morgan fingerprint generation and Tanimoto similarity calculation can be done by Rdkit package [30].
- *Property of generated molecules*, i.e., Property(*Y*), includes QED-score, DRD2-score, and LogP-score, evaluated using Rdkit package [30] or chemical rule.
- *Success Rate (SR)* considers both similarity constraint and property improvement. Following [13], [14], [23], we design a criteria to judge whether it satisfied these two aspects: (a) Input and generated molecules are similar enough, sim(*X*, *Y*) ≥ *λ*_1_; (b) improvement are big enough, i.e., property(*Y*) − property(*X*) ≥ *λ*_2_ or property(*Y*) ≥ *λ*_3_. The selection of *λ*_1_ and *λ*_2_ (or *λ*_3_) depend on datasets. Following [13], [14], [37], [41], for QED and DRD2 dataset, when the similarity between input and generated molecule is greater than 0.3 and chemical property (QED, DRD2) of generated molecule is greater than 0.6, we regard the target molecule as a success. For LogP dataset, when the similarity between input and generated molecule is greater than 0.4 and LogP improvement is greater than 0.8, we regard the target as a success.
- *Novelty* is defined as the percentage of generated molecules that does not appear in training set. We also need to do canonical operation to convert the generated molecule into canonical SMILES.
- *Run time & Model Size*. We also report training run time in hours and the model size. Worth to mention that during the inference (generating molecule) procedure, MOLER do not require extra computation, so inference time is about the same as the generative model baselines. MOLER does not require extra parameters compared with original generative models, so JTVAE + MOLER and JTVAE have the same model size, VJTNN + MOLER and VJTNN have the same size, CORE + MOLER and CORE have the same model size.

### 4.6 Experimental Details for Reproducibility

In this section, we provide the implementation details required for reproduction of experimental results.

#### Features.

First we discuss the features used as input for encoders, including both node and edge features. Following [12], [13], [18], in the (molecular) graph message passing network, the initial atom features include its atom type, degree, its formal charge and its chiral configuration. Bond feature is a concatenation of its bond type, whether the bond is in a ring, and its cis-trans configuration. In the scaffolding tree-based encoder, we represent each substructure with a neural embedding vector, which is similar to word embedding. Both tree and graph decoder leverage the same feature setting as encoders.

#### Hyperparameter.

We follow most of the hyperparameter setting of JTVAE [12], VJTNN [13] and CORE [18] when running JTVAE+MOLER, VJTNN+MOLER and CORE +MOLER, respectively. For all these baseline methods and datasets, the maximal epoch number is set to 10; the batch size is set to 32. The initial learning rate is set to 1*e*^−3^ with the Adam optimizer. Every epoch learning rate is annealed by 0.8. Convergence criteria is checked every epoch measured by success rate on the validation set. When the success rate of the current epoch is lower than the previous epoch, we claim that the model converges and terminate the learning procedure. Following VJTNN and CORE, the discriminator in adversarial training is a three-layer fully-connected network with latent dimension 300 and LeakyReLU function is leverage as activation. For JTVAE, VJTNN, CORE, and their MOLER variants, the depth of tree encoder and graph encoder are set to 6 and 3, respectively. The dimension of hidden state for both (molecular) graph message passing network (GMPN) and junction tree message passing network (JTMPN) are set to 300. All the MPN uses tanh activation and mean function as readout function. In decoder network, all the hidden layer uses ReLU function as activation. MOLER does not bring extra learnable parameters. According to empirical study, the weight of individual MOLER plays a particularly important role in performance. It’s regarded as a gradient-free optimization problem, we resort to Gaussian Process [35], as described in Section 3.5. Regarding both similarity reward and size deviation penalty, the search space of weight ranges from 1*e*^−4^ to 1*e*^−2^.

#### Hardware and Software Configuration.

All the experiments are run on an Intel Xeon E5-2690 machine with 256G RAM and 8 NVIDIA Pascal Titan X GPUs. Our method is implemented by Python 2.7 and Pytorch 0.4.0. RDKit version has to be later than 2017.09.

### 4.7 Q1: MOLER can Improve Deep Generative Models

**Fig. 3.**
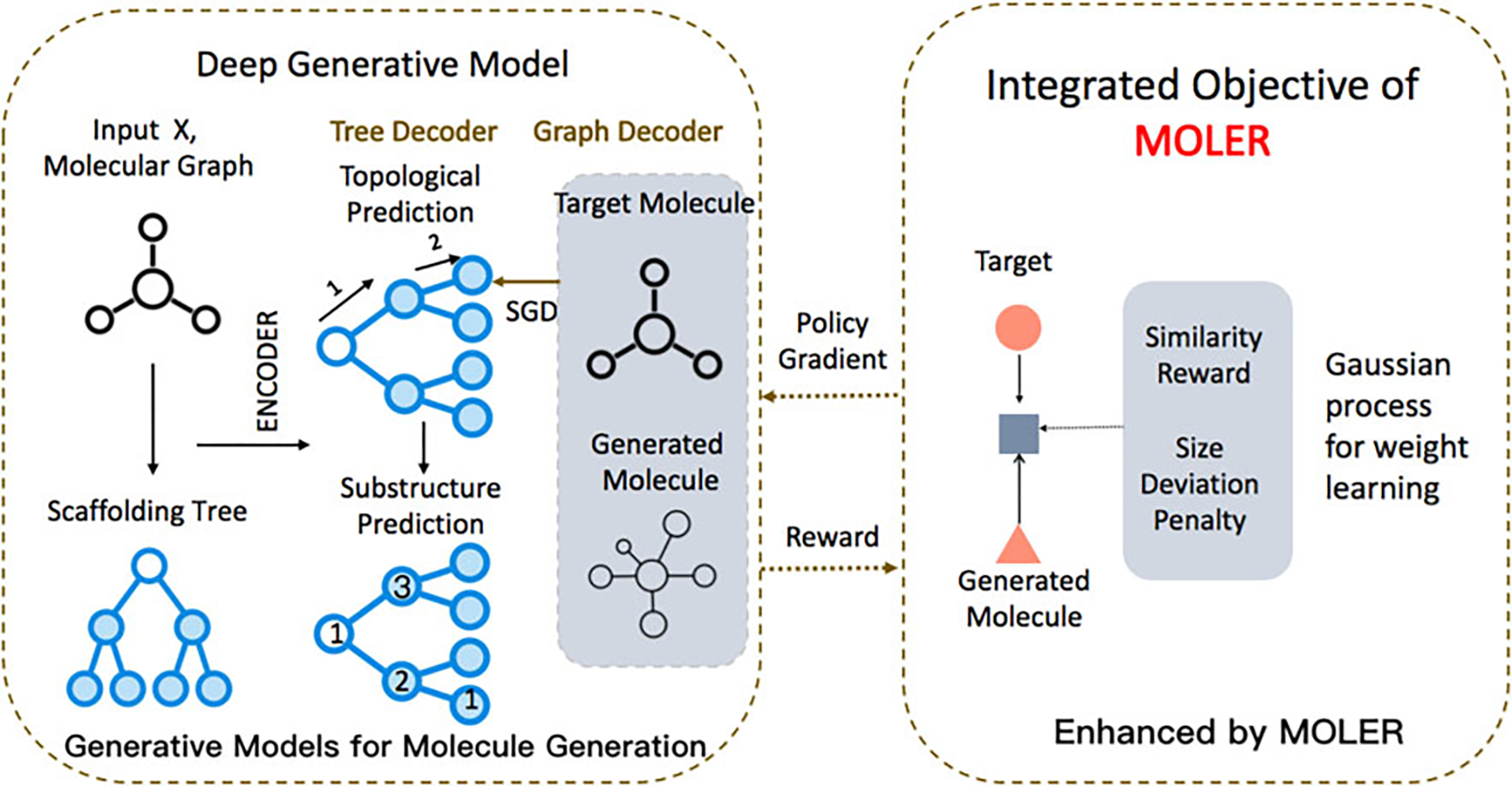
Results measured by novelty, training time, model size and size deviation of generated molecule. (a) All methods have ~ 100% novelty scores, and (b-c) MOLER variants do not cost much more computational cost in training time, and their model sizes are compared to the original generative models. (d) MOLER can reduce the size deviation of generated molecules.

First, we demonstrate that MOLER can improve the state-of-the-art generative model methods on all the three datasets. The results (in terms of similarity, property improvement, and success rate) are reported in Table 4. We found that MOLER variants (JTVAE+MOLER, VJTNN+MOLER, CORE+MOLER) provides significant and consistent improvement across different generative models up to 19%. For other metrics, in Fig. 3, we use bar charts to show the results of various methods on various datasets. MOLER along with other methods can maintain high novelty in the generated molecules in Fig. 3d; MOLER will not cost many extra computational costs in training time and model size as shown in Figs. 3e and f; Regarding running time, we observe that JTVAE-based methods (JTVAE and JTVAE+MOLER) takes a longer time to converge when optimizing LogP score. There are mainly three reasons. (i) When we use JTVAE+MOLER to optimize LogP, we observe during the early iterations, the generated molecules differ a lot from the target ones, the MOLER objective is not stable. Thus it costs more time. (ii) LogP is more sensitive to the local structure [38] compared QED and DRD2 and hard to optimize. We observe that all the methods cost a longer time when optimizing LogP. (3) At the same time, compared with VJTNN, the neural architecture of JTVAE is less finer-grained. Specifically, it does not have adversarial learning module and attention mechanism in the decoder, which may cause a longer time to converge. Finally, as shown in Fig. 3g, MOLER is able to reduce the size deviation, measured by the variance of size(X)-size(Y) on the test set.

#### Case Study.

**Fig. 4.**
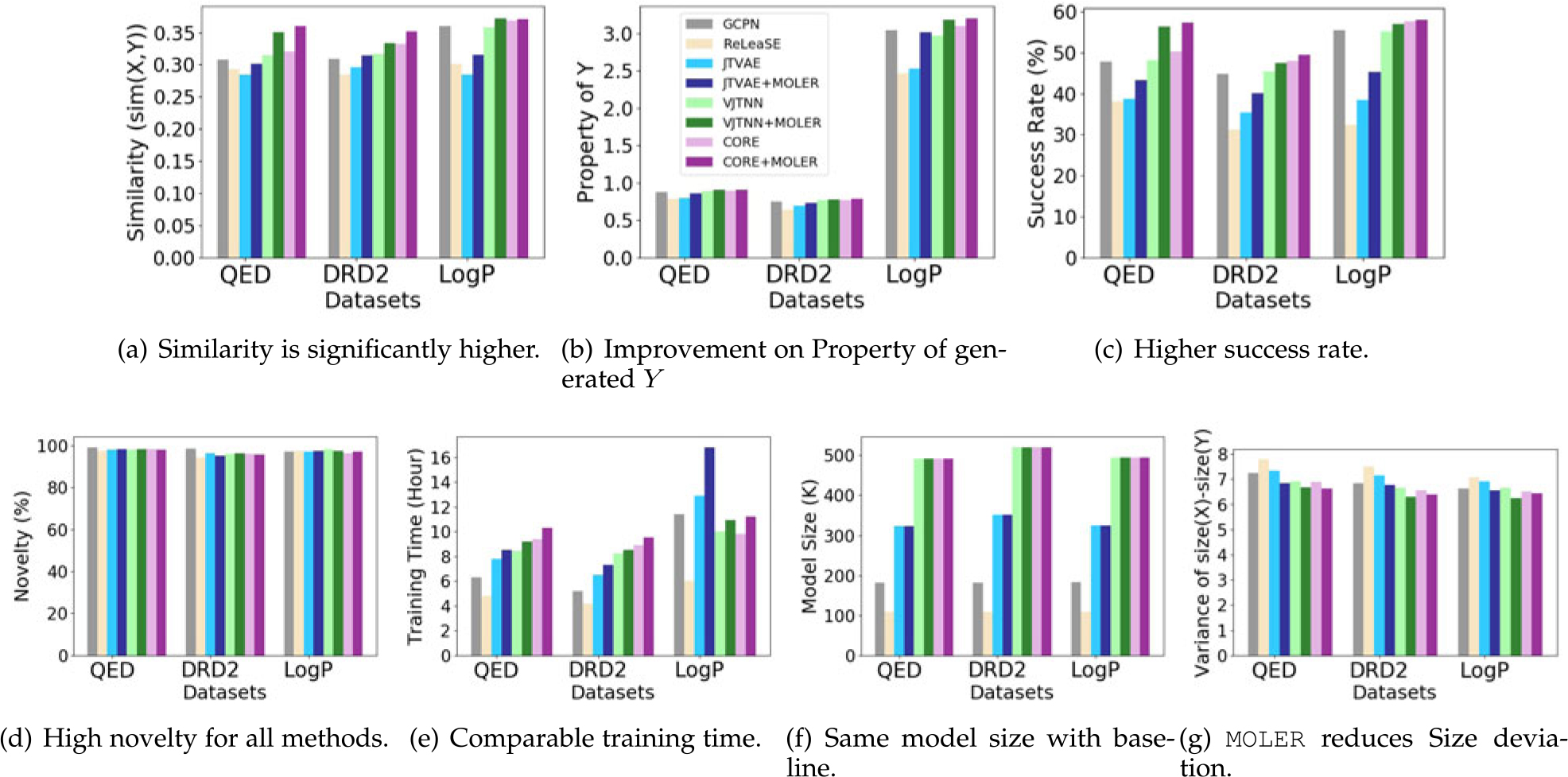
A case study on QED dataset, where VJTNN generate a small molecule with poor performance, VJTNN+MOLER generates a molecule that achieves much better similarity and QED score.

Moreover, we provide an example in Fig. 4, where VJTNN generate a small molecule with poor performance. In contrast, JTVAE+MOLER and VJTNN+MOLER generate target molecules that achieve high similarity and much more improved QED score for the same input molecule.

### 4.8 Q2: The Positive Effect of Similarity Reward

Next, we present the effect of similarity reward defined in Equation (10) (Section 3.2), and show how we set the similarity reward.

In practice, to accelerate the optimization procedure [32], [33], we want to make the average *R*(*X*, *Y*) to be close to 0, so we subtract its average from the original value. For example, in similarity reward, the reward is

(24) R(X,Y)=sim(X,Y)−C,

where C∈ℝ is a hyperparameter, we want it to be close to the average of all the similarity value.

Two hyperparameters play crucial role in the empirical performance of similarity reward: (1) the weight of similarity reward in the whole objective $w_{\text{sim}}\in {\mathbb{R}}_{+}$ in Equation (10); (2) hyperparameter in similarity reward C∈ℝ in Equation (24). We search the weight of similarity reward *w*_sim_ from {1*e*^−4^, 3*e*^−4^, 1*e*^−3^, 3*e*^−3^, 1*e*^−2^}. For hyperparameter in similarity reward *C*, we want it to be close to the average of similarity value. During the learning procedure, the similarity would increase from 0 to about 0.3–0.4. So we search *C* from {0, 0.1, 0.2, 0.3, 0.4}. We use grid search to find the optimal combination of hyperparameters. For all the possible combinations, similarity and property of generated molecules are shown in Table 5. *w*_sim_ = 0 corresponds to baseline method (VJTNN) that don’t use similarity reward. We can find that most of the (*w*_sim_, *C*) combinations can outperform baseline method, validating the effectiveness of adding similarity reward.

**Fig. 5.**
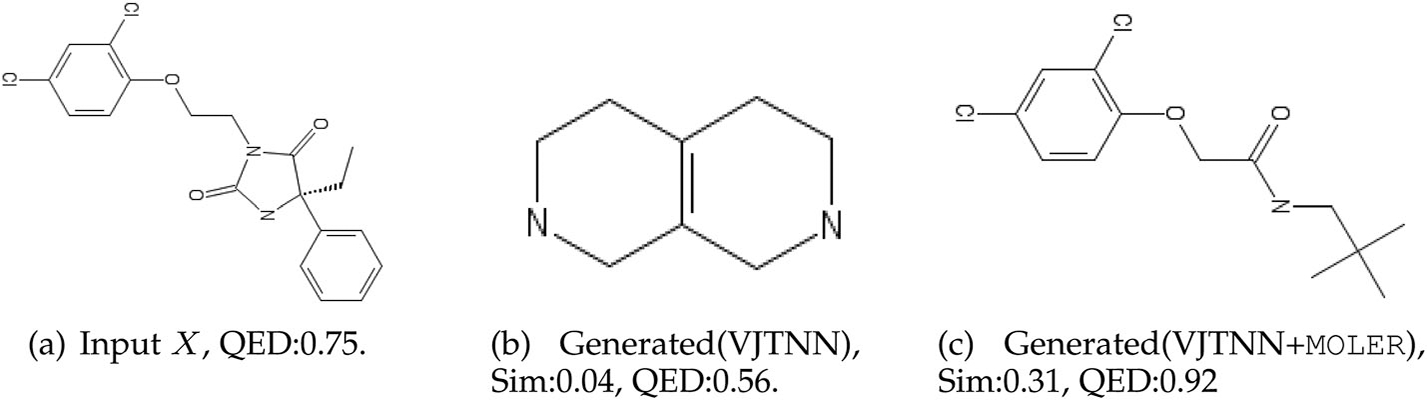
Selection of (*w*_sim_, *C*) on QED dataset for similarity reward (Section 3.2). We find (i) *w*_sim_ = 1*e*^−3^ and *C* = 0.3 performs best among all the hyperparameters, especially on improvement of similarity; (ii) within a reasonable range, the similarity would increase when weight *w*_sim_ increase. However, too large *w*_sim_ would degrade the performance, even worse than baseline method (VJTNN, dashed line).

In addition, we show more visualization results in Fig. 5. For each weight *w*_sim_ ∈ {1*e*^−4^, 3*e*^−4^, 1*e*^−3^, 3*e*^−3^, 1*e*^−2^}, we show the change of performance (both similarity and property) with different *C*s (in Equation (24)) in Figs. 5a and 5b. Also, for each *C* ∈ {0, 0.1, 0.2, 0.3, 0.4}, we show the change of performance with various *w*_sim_ in Figs. 5c and 5d. We find that (i) *w*_sim_ = 1*e*^−3^ performs best among all the hyperparameters, especially on improvement of similarity; (ii) When *C* = 0.3, MOLER achieve the best performance; (iii) within a reasonable range, the similarity would increase as we increase the weight *w*_sim_; (iv) too large *w*_sim_ would degrade the performance, even worse than baseline method (VJTNN).

For QED dataset, the optimal combination of hyperparameters is *w*_sim_ = 1*e*^−3^ and *C* = 0.3, as mentioned above. It is also validated to be optimal in DRD2 dataset. For LogP dataset, the optimum is *w*_sim_ = 1*e*^−3^ and *C* = 0.4.

### 4.9 Q3: The Positive Impact of Size Deviation Penalty

Next, we demonstrate the effect of the size deviation penalty on the quality of generated molecules. The selection of reward weight *w*_size_ and the reward function *g* (Equation (14)) are important to the performance. We also compare the performance of different size deviation penalty functions, where *x*, *y* represent the size of input *X* and generated *Y*, respectively.

- *g*_1_, reward function that discourage generating small molecule. It set a global threshold *S* for all the molecules, so it doesn’t depend on the input molecule *X*. It is defined as
(25) $$g_{1}(x,y)=\{\begin{array}{ll} 0, & y\geq S,\\ S-y, & y<S, \end{array}$$
  it assigns large penalty when size of *Y* is small, We set *S* = 10 because it achieve best performance. The average number of substructures in training set is around 14, as shown in Table 3.
- *g*_2_, reward function that discourage generating large molecule. Similar with *g*_1_, it set a global threshold *T* for all the molecules and is defined as
(26) $$g_{2}(x,y)=\{\begin{array}{ll} 0, & y\leq T,\\ u-T & u>T, \end{array}$$
  which returns large penalty for large molecule. *T* = 17 achieve best performance.
- *g*_3_, reward function that discourage generating small molecule compared with the size of input molecule *X*. It is defined as
(27) $$g_{3}(x,y)=\left\{\begin{array}{ll} 0, & y\geq x-\epsilon ,\\ |x-y|-\epsilon , & y<x-\epsilon , \end{array}\right.$$
  it returns a big penalty when the size of *Y* is much smaller than the size of *X*.
- *g*_4_, reward function that discourage generating large molecule. Similar with *g*_3_, It depends on the size of input molecule *X* and is defined as
(28) $$g_{4}(x,y)=\{\begin{array}{ll} 0, & y-x\leq \epsilon ,\\ |x-y|-\epsilon , & y-x>\epsilon , \end{array}$$
- *g*_5_ (*g*_5_ = *g* in Equation (14) in Section 3.3) can be seen as a combination of *g*_3_ and *g*_4_. It will generate penalty when the size of generated *Y* deviate significantly from the input molecule *X*. It is defined as
(29) $$g_{5}(x,y)=\{\begin{array}{ll} |x-y|-\epsilon , & |x-y|>\epsilon ,\\ 0, & |x-y|\leq \epsilon . \end{array}$$

For *g*_3_, *g*_4_ and *g*_5_, *ϵ* = 3 perform best among *ϵ* ∈ {1, 2, 3, 4, 5} on QED, so it’s fixed to 3.

**Fig. 6.**
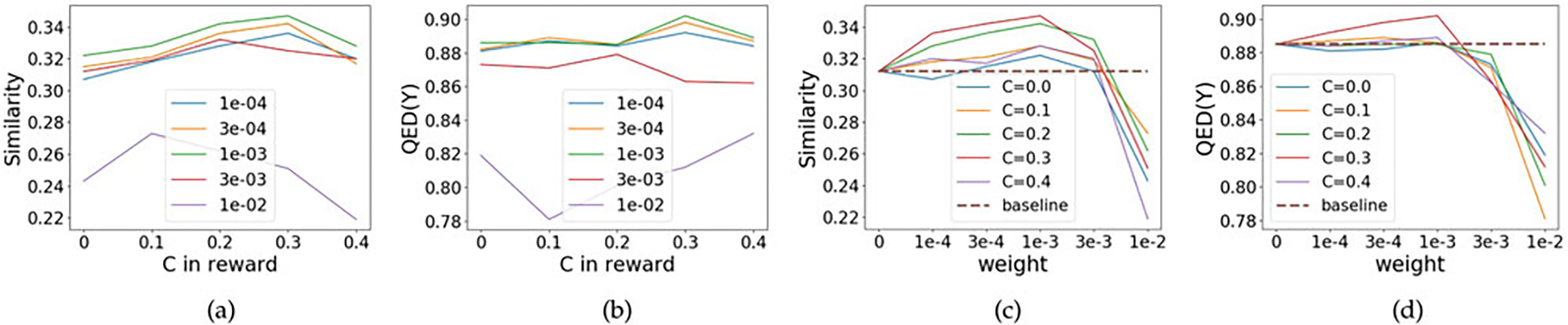
Empirical effect of different size deviation penalty function (from *g*_1_ to *g*_5_, Section 4.9) for size deviation penalty on QED using VJTNN+MOLER. The dash lines correspond to baseline VJTNN (weight of reward is set to 0). *g*_5_ and *w*_size_ = 1*e*^−3^ obtain the best performance among all selections.

For each size deviation penalty *g*, we plot the change of performance (both similarity and property) with various weight *w*_size_ in Fig. 6 on the QED dataset. We find that (i) *g*_5_ (which is represented in Section 3.3) and *w*_size_ = 1*e*^−3^ achieve the best performance; (ii) most of the hyperparameter setting would outperform VJTNN (baseline). Therefore, the effect of size deviation penalty is positive. Moreover, this selection is also validated to be optimal on both DRD2 and LogP datasets.

## 5 Conclusion

In this paper, we have proposed to incorporate molecule level reward function (MOLER) into deep generative models for molecule optimization. Specifically, we have designed two molecule reward functions motivated by some empirical observations. The first one is the similarity reward to encourage the generated molecule to be similar to the input one. Another reward is to control the size of the generated molecule explicitly. MOLER provides a general and flexible framework that can incorporate various reward functions to specify different aspects of generated molecules based on any deep generative models for molecule optimization. Policy gradient is applied to optimize the reward objective, and it wouldn’t cause too much extra computational cost compared with deep generative models. Thorough empirical studies have been conducted on several real molecule optimization tasks to validate the effectiveness of MOLER.

In the future, we want to explore the following directions: (1) designing more reward functions that can improve the molecule optimization procedure; (2) decomposing molecule level reward into substructure level to make the learning procedure easier and more efficient.

## Figures and Tables

**TABLE 1:** Empirical Results of Two Deep Generative Models on Two Molecule Optimization Tasks, Improving QED and DRD2 Score

Dataset	Method	All Test Set		Small Molecule		Large Molecule	
		Similarity	Property	Similarity	Property	Similarity	Property

QED	JTVAE [12]	0.298	0.804	0.142	0.542	0.231	0.792
	VJTNN [13]	0.311	0.885	0.178	0.602	0.283	0.818
DRD2	JTVAE	0.299	0.709	0.164	0.501	0.243	0.621
	VJTNN	0.315	0.765	0.170	0.579	0.296	0.744

QED and DRD2 are important chemical properties for drug molecules. Small molecule includes the generated molecules with less than eight substructures while large molecule includes the ones generated with more than 18 substructures. “Similarity” is the similarity between input and generated molecule. “Property” (either QED or DRD2) is the average property score of the generated molecules. Both scores are basic metrics for molecule optimization, ranging from 0 to 1, and higher is better. We report the average results on test set. We find that (1) similarity score has more improvement space compared with property scores; (2) performance degradation on either small or large molecules.

**TABLE 2:** Notations Used in the Paper

Notations	short explanation

(*X*, *Y*)	input-target molecule pair
*S*	substructure set *S* (a.k.a. vocabulary)
*V/E*	set of vertex(atom) / edge(bond)
*G* = (*V*, *E*)	molecular graph
${𝒯}_{G}=(𝒱,ℰ)$	scaffolding tree of graph *G*, junction tree [12]
$T_{G}$	scaffolding tree of *G* without substructure, output of topological prediction, as shown in Fig. 2
*N*(*v*)	set of neighbor nodes of vertex *v*
**e***_v_*/**e***_uv_*	feature vector for node *v* / edge (*u*, *v*)
$m_{uv}^{\left(t\right)}$	message for edge (*u*, *v*) at the *t*th iteration
*T*	Depth of Message Passing Network
$Z_{i}^{G}/z_{i}^{𝒯}$	embedding of node *i* in G/𝒯, $Z^{G}=\left\{z_{1}^{G},\ldots \right\}$
$h_{i_{t},j_{t}}$	message vector for edge (*i_t_*, *j_t_*)
**d** *_G_*	Embedding of Graph *G*
$p_{t}^{\text{topo}}$	topological prediction score at the *t*th step
$q_{t}^{\text{sub}}$	substructure prediction distribution at the *t*th step
*f_i_*(·), *i* = 1, …, 6	parameterized neural networks
sim(*X*, *Y*) ∈ [0, 1]	Similarity between *X* and *Y*
size(*X*)	number of substructure in *X*
*θ*	all learnable parameters in generative model
*π_θ_*(*Y*|*X*)	generative model parameterized by *θ*
*g*_1_(·), *g*_2_(·)	fully-connected neural network

**TABLE 3:** Statistics of all the Three Datasets: DRD2, QED, LogP

Dataset	# Training Pairs	# Valid Pairs	# Test	Vocab Size	Avg # Substructures	*η*_1_

QED	84,306	4,000	800	780	14.99	0.4
DRD2	32,404	2,000	1,000	967	13.85	0.4
LogP	94909	5,000	800	785	14.30	0.4

*Here vocabulary is the set of all substructures. “Avg # Substructures” is the average number of substructures per molecule. η*_1_ *is the threshold for similarity constraint defined in Equation (23). QED, DRD2, LogP are not only the chemical properties in this paper. They are also used to name the datasets, e.g., QED dataset is used to learn a model for improving the QED score of a molecule.*

**TABLE 4:** MOLER-Based Methods Outperform Deep Generative Methods (JTVAE [12], VJTNN [13] and CORE [18]) and RL Methods (GCPN [7], ReLeaSE [20])

Method	Similarity			Property Improvement			Success Rate (%)		
	QED	DRD2	LogP	QED	DRD2	LogP	QED	DRD2	LogP

GCPN	0.308	0.309	0.360	0.877	0.745	3.041	47.71	44.81	55.43
ReLeaSE	0.293	0.284	0.302	0.783	0.643	2.465	38.02	31.21	32.38
JTVAE	0.299	0.300	0.285	0.791	0.693	2.532	38.74	35.43	38.43
JTVAE+MOLER	0.302	0.314	0.315	0.858	0.732	3.015	43.20	40.01	45.24
improvement	+1.07%	+4.77%	+10.41%	+8.47%	+5.63%	+19.08%	+11.51%	+12.93%	+17.72%
VJTNN	0.315	0.316	0.358	0.886	0.764	2.972	48.16	45.38	55.15
VJTNN+MOLER	0.351	0.334	0.372	0.904	0.778	3.182	56.32	47.39	57.01
improvement	+11.61%	+5.56%	+3.94%	+2.03%	+1.83%	+7.07%	+16.94%	+4.43%	+3.37%
CORE	0.321	0.333	0.369	0.895	0.769	3.100	50.26	47.91	57.64
CORE+MOLER	0.360	0.352	0.371	0.910	0.782	3.199	57.32	49.47	57.93
improvement	+12.11%	+5.58%	+.41%	+1.68%	+1.69%	+3.19%	+14.05%	+3.26%	+.50%

MOLER-based methods achieved the best performance in all settings, especially CORE+MOLER, which seems the most competitive. MOLER provides significant and consistent improvement across different generative models up to 19%. In each column, we highlight the best performance using bold font.

**TABLE 5:** Empirical Performance of Adding Only Similarity Reward for Different Hyperparameter on QED Dataset

weight *w*_sim_ *C* in reward	0	1*e*^−4^	3*e*^−4^	1*e*^−3^	3*e*^−3^	1*e*^−2^

0	0.314, 0.886	0.307,0.881	0.315,0.882	0.322,0.886	0.312,0.873	0.243,0.819
0.1		0.318,0.887	0.321,0.889	0.328,0.886	0.319,0.871	0.273,0.781
0.2		0.328,0.884	0.336,0.885	0.342,0.885	0.332,0.879	0.262,0.801
0.3		0.336,0.892	0.342,0.898	**0.347,0.902**	0.325,0.863	0.251,0.812
0.4		0.320,0.884	0.317,0.887	0.328,0.889	0.320,0.862	0.219,0.832
ADAPT		0.338,0.888	0.340,0.893	0.345,0.897	0.335,0.863	0.298,0.813

*Average* Sim(*X*, *Y*), QED(*Y*) *are reported. We use grid search to find the best hyperparameter combination for similarity reward. The optimal selection is w*_sim_ = 1*e*^−3^, *C* = 0.3, *reaching the best similarity and property improvement at the same time.*

## Footnotes

1. Such a scaffolding tree is also named as a junction tree in [12], [13].

2. *T* is a hyperparameter, similar to that in Graph Convolutional Network [27].

3. ZINC database (https://zinc.docking.org) is a curated collection of commercially available chemical compounds prepared especially for virtual screening [39].

4. ZINC database is publicly available at https://zinc.docking.org.
